# Self-Powered Memristive Systems for Storage and Neuromorphic Computing

**DOI:** 10.3389/fnins.2021.662457

**Published:** 2021-03-31

**Authors:** Jiajuan Shi, Zhongqiang Wang, Ye Tao, Haiyang Xu, Xiaoning Zhao, Ya Lin, Yichun Liu

**Affiliations:** ^1^Key Laboratory for Ultraviolet Light-Emitting Materials and Technology (Northeast Normal University), Ministry of Education, Changchun, China; ^2^School of Science, Changchun University of Science and Technology, Changchun, China

**Keywords:** neuromorphic computing, memristor, self-powered, artificial intelligence, nanogenerator

## Abstract

A neuromorphic computing chip that can imitate the human brain’s ability to process multiple types of data simultaneously could fundamentally innovate and improve the von-neumann computer architecture, which has been criticized. Memristive devices are among the best hardware units for building neuromorphic intelligence systems due to the fact that they operate at an inherent low voltage, use multi-bit storage, and are cost-effective to manufacture. However, as a passive device, the memristor cell needs external energy to operate, resulting in high power consumption and complicated circuit structure. Recently, an emerging self-powered memristive system, which mainly consists of a memristor and an electric nanogenerator, had the potential to perfectly solve the above problems. It has attracted great interest due to the advantages of its power-free operations. In this review, we give a systematic description of self-powered memristive systems from storage to neuromorphic computing. The review also proves a perspective on the application of artificial intelligence with the self-powered memristive system.

## Introduction

Brain-like artificial neural networks (ANNs) are currently gaining extensive attention as an evolving technology for artificial intelligence, enabling self-learning, speech recognition, and pattern recognition (Jiang et al., 2017; Van and Bohte, 2017; Peng et al., 2019; George et al., 2020; Kumar et al., 2020). Memristor possesses the significant advantage of resistance-tunable features for behaving like a synapse and is universally considered to be the emerging building blocks of brain-like ANNs (Wang et al., 2016; Li C. et al., 2018; Liu et al., 2018; Yan et al., 2018, 2019a,b; Yoon et al., 2018; Zhao et al., 2020). Some progress has been made in emulating synapse behavior and constructing hardware ANNs with memristors devices (Wang et al., 2012; Kim et al., 2015; Zhang et al., 2017; Hu et al., 2018; Ntinas et al., 2018). Prezioso et al. (2015) experimentally demonstrated an artificial neural network using metal-oxide based memristors integrated into a dense, transistor-free crossbar circuit. Yang’s group used memristor-based ANNs to demonstrate the capabilities of artificial intelligence, including online reinforcement learning, unsupervised learning using fully memristive networks, and convolutional memristor networks, all of which are significant developments in memristor-based brain-like ANNs (Wang et al., 2018, 2019a,b). However, when building larger-scale brain-like or implantable ANNs systems, the proportional increase of energy consumption and power-supply circuit complexity are inescapable problems (Tao et al., 2020). Research efforts have therefore been devoted to the development of energy-efficient memristive systems (Tao et al., 2017; Zhao X. et al., 2017; Jang et al., 2018; Zidan et al., 2018; Choi et al., 2020; Lübben et al., 2020). Traditional approaches for achieving ultra-low-power memristors include optimizing the switching operation, for example reducing switching current/voltage, and increasing switching speed, and so forth (Li Y. et al., 2018; Tao et al., 2018; Qi et al., 2020; Tian et al., 2020; Tseng et al., 2018).

The pursuit of high energy efficiency memristor devices continues and a newly developed self-powered technology that can harvest environmental energy of various kinds to drive functional units shows promise. This self-powered technology completely decouples the dependency on an external power-supply system (Zhao et al., 2015; Ding et al., 2017; Tan et al., 2017; Zhao F. et al., 2017; Xu et al., 2019). It has been extensively investigated in several prominent applications, in fields such as implantable medical devices, the aerospace industry, and remote monitoring (MacVittie and Katz, 2014; Sun et al., 2017; Xu et al., 2018; Yang Z. et al., 2020). These reports indicate that self-powered technologies can usually be divided into piezoelectric, triboelectric, fluidic-electric, pyroelectric, photovoltaic, and moisture-electric effects (Yin et al., 2014; Liu et al., 2016; Shao et al., 2017; Luo et al., 2018; Chandrasekaran et al., 2019; Stewart et al., 2020). Integrating these self-powered systems with various functional devices, power-free electronics, such as self-powered generators, self-powered sensors, self-powered detectors, and self-powered motors have been successfully developed (Lee et al., 2020; Wang Y. et al., 2020; Zhang and Yeow, 2020). Inspired by the above power-free electronics systems, memristors, the elementary unit of ANNs, have also been incorporated with self-powered technologies. Table 1 shows recent reports about self-powered memristive systems, from which we can see that by introducing different self-powered technologies, multiple self-powered electronic devices can be developed, a research hotspot currently being explored by many researchers.

**TABLE 1:** Self-powered memristor series for various applications.

Study	Memristive component	Self-powered component	Potential application
MacVittie and Katz (2014)	ECM memristor	Biofuel cell	Self-powered information processing
Tan et al. (2015)	VCM memristor	Photoelectric effect	Self-powered storage
Kim et al. (2016)	VCM memristor	Piezoelectric nanogenerator	Self-Powered storage
Lee et al. (2017)	VCM memristor	Piezoelectric nanogenerator	Self-Powered storage
Zhao F. et al. (2017)	VCM memristor	Moisture-electric nanogenerator	Self-Powered storage
Sun et al. (2017)	VCM memristor	Triboelectric nanogenerator	Self-powered smart skin
Kim et al. (2017)	VCM memristor	Piezoelectric nanogenerator	Self-powered artificial synapse
Zhou G. et al. (2019)	ECM memristor	Moisture-electric nanogenerator	Self-powered storage
Liu et al. (2019)	Organic field effect transistor	Triboelectric nanogenerator	Self-powered tactile system
Chen et al. (2019)	Ion gel-gated transistor	Piezoelectric nanogenerator	Self-powered sensory synapse
Zhou F. et al. (2019)	VCM memristor	Photoelectric effect	Self-powered neuromorphic vision
Liu et al. (2020a)	Field effect transistor	Triboelectric nanogenerator	Self-powered auditory pathway
Tao et al. (2020)	VCM memristor	Moisture-electric nanogenerator	Self-powered reading
Liu et al. (2020b)	Field effect transistor	Triboelectric nanogenerator	Self-powered sensory memory
Yang X. et al. (2020)	Halide perovskite memristors	Photovoltaic nanogenerator	Self-powered retina system
Yang Z. et al. (2020)	VCM memristor	Piezoelectric nanogenerator	Self-powered pressure sensor

Generally, self-powered memristors structurally consist of an electric nanogenerator and a memristive unit that connect each other in series. The output amplitude from the nanogenerator is sufficient to drive the memristor, thus the integrated self-powered memristor devices possess an ability that could operate normally without an external power supply. This kind of power-free electronic system has enormous application potentials in some special environments, such as the aerospace industry and implantable medicine. Therefore, it would be very significant and interesting to develop self-powered memristive systems. The objective of this paper is to give a review of the updated research progress of recently self-powered memristive systems from storage application to neuromorphic computing. We also provide some suggestions and optimization methods for the development of artificial intelligence with self-powered memristive systems.

## Self-Powered Memristive Systems for Storage Application

Digital memristor, the resistance value of which can be switched between a high resistance state (HRS) and low resistance state (LRS), is a very promising candidate for next generation non-volatile memory due to its superior performance and simple structure (Sakib et al., 2017; Zhao et al., 2019; Wang Z. et al., 2020). However, as a passive electronic device, the memristor has to be driven by an external bias voltage. A promising way of addressing this could involve combining the memristor with a self-powered technology that can facilitate a completely power-free operation. A detailed discussion of recent research results in relation to the self-powered memristive storage system is outlined below.

At present, humidity-powered systems are widely reported (Zhao et al., 2015; Ding et al., 2017; Zhao F. et al., 2017), mainly because water and vapor are the most sufficient resources on the earth. Imitating the driving force of water vapor evaporation along a tree trunk, Xue et al. (2017) successfully fabricated a humidity-powered electric generator using nanostructured amorphous carbon materials. This approach is exciting as it uses evaporation from a centimeter-sized carbon-based material sample that could reliably generate sustained voltages of up to 1 V for at least 163 h under humid conditions. Based on this highly efficient nanogenerator, Zhou et al. fabricated a self-powered porous carbon-based memristor device (Zhou G. et al., 2019). They integrated a memristor device with Ag/amorphous carbon/Ag sandwich structure and three aforementioned water-evaporation-induced nanogenerators using wires in series. The memristor device could be driven by the carbon-based nanogenerator, and thus operate completely power-free. In addition, the output ability of this nanogenerator could be linearly increased when connecting multiple nanogenerator cells in series. This self-powered memristor device has the advantages of being sustainable, renewable, environmental-friendly and it meets the development direction and use requirements of green electronics. The above experiment indicates that the integration of multiple devices is a promising approach for multifunctional power-free intelligent electronics.

**FIGURE 1:**
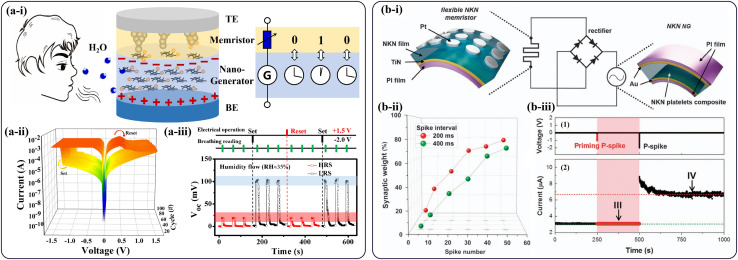
**(a-i)** Schematic diagram of the moisture-powered memristor with a W/WOx/OAC/Pt structure and its moisture-powered reading operation. The OAC and WO_x_ films act as the nanogenerator and memristor layer, respectively. **(a-ii)** Typical I-V curves of the integrated memristor device. **(a-iii)** Power-free reading of the HRS/LRS through human breath after reversible RS operations (Tao et al., 2020). **(b-i)** Schematic diagram of an NKN-based memristor operated by the NKN NG. **(b-ii)** Variations of the synaptic weight with respect to the spike number and interval. **(b-iii)** (1) Priming P-spike and P-spike applied to the NKN memristor and (2) current of the NKN memristor (Kim et al., 2017).

Furthermore, the existing self-powered memristive systems involve the structural integration of a nanogenerator and a memristor in series. These are two independent systems owing to their different operation mechanisms. It would be interesting to develop a self-powered memristive system in which these two systems are integrated with correlated mechanisms. Inspired by previous work, a moisture-powered memristive system with a close mechanism correlation were fabricated with a double-layer stack of a WO_x_ memristor layer and an oxygen-plasma-treated amorphous carbon (OAC) nanogenerator layer by Tao et al. (2020), as is shown in Figure 1a-ii. The resistance switching mechanism of the integrated system can be attributed to the formation/rupture of several tiny V_o_-based CFs in the memristor. When a negative bias voltage is applied on the top electrode, the oxygen ions in the WOx film are driven into the OAC film, resulting in the LRS with the oxygen-deficient CFs. The increased oxygen content in the OAC film will objectively give rise to an increase in oxygen gradient. The output voltage of this integrated moisture-powered memristor will therefore increase, and vice versa. The integrated self-powered memristor device was capable of good switching performance (Figure 1a-i). An 8 × 4 cell array was constructed to demonstrate a practical application of the moisture-powered reading operation. The output signals of the HRS and LRS are, respectively represented by the binary values 1 and 0 (Figure 1a-iii), and the pre-programmed single letters can be simultaneously displayed by the standard eight-bit code of ASCII under the circumstance of human-breathing. Moreover, by controlling the number of mobile oxygen species, the moisture-powered memristor revealed the capability of multi-level storage and selective reading function using a digital comparator to differentiate digital signals. The integrated self-powered memristor device, especially with a mechanism correlation concept, may open the pathway to the development of novel self-powered electronics.

Except for moisture-powered memristive systems, several other types of self-powered technologies have also introduced, as shown in Table 1. For example, Kim et al. (2016) have fabricated a low-temperature-grown amorphous (Na_0.5_K_0__.__5_)NbO_3_ piezoelectric nanogenerator (NKN NG). They have successfully constructed a self-powered memristor by using wires to connect this NKN NG and an NKN-based memristor device. The NKN NG has a large open-circuit output voltage of ∼2.0 V and a short-circuit output current of ∼40 nA, which serves as the power supply to drive the memristor device to operate. Stable switching properties with a large ON/OFF ratio of 102 were obtained. In addition, to verify the biocompatibility of component materials, they also conducted cell-viability tests on NKN thin films, showing the potential application of NKN films to implantable biomedical devices.

Some researchers have also introduced light to drive the memristor device, which can also be classified as a kind of self-powered technology. For example, Chai’s group demonstrated a novel optoelectronic memristor based on a Pd/MoO_x_/ITO sandwich structure. The introduction of optical operation could not only simplify the circuitry of image processing but also reduce power consumption. These light-tunable memristor cells could be used as building blocks for the application of neuromorphic visual pre-processing (Zhou F. et al., 2019). Tan et al. (2015) used the modulation mechanism of the band bending at the CeO2-x/AlOy/Al region to show a light-writeable/electrically erasable memristive device, integrating demodulating, arithmetic and memory functions.

## Self-Powered Memristive Systems for Neuromorphic Computing

As one promising device for artificial synapses, an analog memristor can realize the multiple functions of biological synapses, which can further realize the construction of ANNs. The collaboration of memristive synapses and different self-power systems can bring some novel and interesting functions.

Usually, the inserted self-powered system can serve as an external signal to realize multiple intrinsic function simulations of artificial synapses. For example, Kim et al. (2017) demonstrated a self-powered artificial synapse system by integrating a flexible (Na_0.5_K_0.5_)NbO_3_ (NKN) memristor and an amorphous (Na_0.5_K_0.5_)NbO_3_ piezoelectric nanogenerator (NKN NG), as is shown in Figure 1b. In their work, a large output voltage of more than −2.0 V was obtained to drive the NKN memristor when the NKN NG was pressed. As is shown in Figure 1b-i, by using an NKN NG to provide priming spikes, they have regulated the spike-time-dependent plasticity (STDP) in the NKN memristor. By controlling the oxygen vacancies movement and shape of the conductive filaments in the NKN memristor, various synaptic behaviors such as a transition from short-term-plasticity (STP) to long-term-plasticity (LTP) and spike-rate-dependent plasticity (SRDP) were realized (Figure 1b-ii). Moreover, they investigated the metaplasticity characteristic of spike-time-dependent plasticity (STDP), which indicates that a priming stimulus before the main actions can modulate the behaviors of synaptic plasticity (Figure 1b-iii). The results indicated that the NKN memristor with the NKN NG has exhibited an excellent synaptic plasticity behavior, which, in an artificial synapse, implements advanced and bio-realistic functions.

Liu et al. (2020b) demonstrated a self-powered artificial sensory memory system by integrating a triboelectric nanogenerator (TENG) and a field effect synaptic transistor (FEST). The voltage spike signal was obtained when an external touch was applied to the triboelectric nanogenerator, which was subsequently transmitted to the gate electrode of the transistor. By utilizing the self-powered artificial sensory memory system, some basic synaptic behaviors such as excitatory post-synaptic current (EPSC) and paired-pulse facilitation (PPF) were well mimicked, and a gradual memory process from sensory memory (SM) and short-term memory (STM) to long-term memory (LTM) were also realized. Furthermore, they realized that a perceptual learning capability of the artificial sensory system and the pattern recognition based on the unsupervised learning process. A series of voltage pulses translated from a training image were applied to a 28 × 28 array as the input signal. After forty thousand times learning, the recognition rate was up to 84%, which is close to the ideal accuracy of the database. Moreover, this self-powered sensory memory system also realized real-time neuromorphic computing with high pattern recognition accuracy.

With the help of some specific nanogenerators, researchers have demonstrated various interesting self-powered applications that can improve the development of power-free artificial intelligence. For example, Liu et al. (2020a) have designed a self-powered artificial auditory pathway actuated by incorporating a triboelectric nanogenerator and a field effect synaptic transistor, emulating the biological auditory functionalities for the first time. Furthermore, they have achieved a self-adaptation artificial auditory pathway with noise adjustable behavior. Recently, Yang’s group designed a self-powered artificial retina perception system by using two-terminal silicon solar cells and an ITO/CsPbBr_2_I/P3HT/Ag memristor device together (Yang X. et al., 2020). In their system, the electrical signal was generated by a light stimulation through the solar cell and transferred to the perovskite-based memristor as a pre-synaptic signal, realizing further information pre-processing functions. Utilizing their self-powered artificial retina perception system, many basic synapse behaviors such as irradiation-time-dependent plasticity (ITDP), irradiation-intensity-dependent plasticity (IIDP), irradiation-wavelength-dependent plasticity (IWDP), and irradiation-frequency-dependent plasticity (IFDP) have been successfully emulated. Moreover, the self-powered artificial retina system can realize the image pre-processing functions of contrast enhancement and noise reduction, which are important for implementing the function of convolutional neural networks (CNNs). Wu’s group has reported a triboelectric nanogenerator-based intelligent neuromorphic tactile sensor (Wu et al., 2019). By inserting a reduced graphene-oxide layer into the friction layer, the device can emulate self-powered neuroplasticity without neuromorphic circuits. The device, with self-powered pressure sensing and learning ability, can serve as a functional element that is suitable in a pressure sensor network of the artificial intelligence system.

## Prospects and Conclusion

Attempts to realize memristor devices that are integrated with various self-powered systems are a major milestone in the field of improving the energy efficiency of memristive systems. This research offers a completely new paradigm for reducing power consumption. From the multiple self-powered memristive systems listed in Table 1, we can see that these systems show huge advantages, especially in some special environments where it is difficult to obtain an external power supply, such as the aerospace industry and implantable medicine, research fields which have attracted more and more attention from researchers in recent years. Most of the existing self-powered memristive systems are based on ideas of structural integration, for example, the series connection of a nanogenerator and a memristor by wires are two independent systems owing to their different operation mechanisms. This impedes the integration level of self-powered memristive systems, restricting applications of this system. It is, therefore, necessary to develop more types of self-powered memristive systems for novel concepts of intelligent electronics.

There are still some challenges that need to be addressed before the practical application of self-powered memristive electronic systems. A major challenge is reducing the size of the self-powered nanogenerator. The configuration structure of various nanogenerators greatly limits their integration with memristor devices, which is a matter of concern for multifunctional applications. In addition, a suitable parameter matching relation between the memristor cell and nanogenerator is significant for realizing multiple functions. Therefore, the second challenge is optimizing the output characteristics of the nanogenerator, including the amplitude of voltage/current and response speed, and the third challenge is reducing the operating power consumption of memristor cells. Furthermore, as mentioned above, the two functional units of self-powered memristive systems (that is, a memristor and a nanogenerator) are connected in series by wires. Therefore, the fourth challenge is a preferable structure integration between memristors and nanogenerators, which will directly affect the integration level of the self-powered memristive systems. The last challenge is the choice of the material of the self-powered memristive system. For example, the materials need to possess good biological compatibility characteristics for applications in the field of implantable medicine, and stable radiation-hardened characteristics for the aerospace industry.

In conclusion, the combination of the memristive device and the self-powered system could be a potential way of realizing multifunctional power-free storage neuromorphic computing, which could satisfy some special needs of novel conceptional artificial intelligence. We believe that such a self-powered memristive system will bring about a brand-new research area that could greatly improve the development of future electronic technologies.

## Author Contributions

JS and YT were responsible for the conception of the review, the literature search, and writing of the manuscript. ZW supervised the selection of the studies and contributed to revision of the manuscript. All authors revised, read, and approved the submitted version.

## Conflict of Interest

The authors declare that the research was conducted in the absence of any commercial or financial relationships that could be construed as a potential conflict of interest.

## References

[B1] Chandrasekaran S. Bowen C. Roscow J. Zhang Y. Dang D. K. Jung E. (2019). Micro-scale to nano-scale generators for energy harvesting: self powered piezoelectric, triboelectric and hybrid devices. *Phys. Rep.* 792 1–33. 10.1016/j.physrep.2018.11.001

[B2] Chen Y. Gao G. Zhao J. Zhang H. Yu J. Yang X. (2019). Piezotronic graphene artificial sensory synapse. *Adv. Funct. Mater.* 29:1900959. 10.1002/adfm.201900959

[B3] Choi S. Yang J. Wang G. (2020). Emerging memristive artificial synapses and neurons for energy-efficient neuromorphic computing. *Adv. Mater.* 32:2004659. 10.1002/adma.202004659 33006204

[B4] Ding T. Liu K. Li J. Xue G. Chen Q. Huang L. (2017). All-printed porous carbon film for electricity generation from evaporation-driven water flow. *Adv. Funct. Mater.* 27:1700551. 10.1002/adfm.201700551

[B5] George D. Lázaro-Gredilla M. Guntupalli J. S. (2020). From CAPTCHA to commonsense: how brain can teach us about artificial intelligence. *Front. Comput. Neurosci.* 14:97. 10.3389/fncom.2020.554097 33192426 PMC7645629

[B6] Hu M. Graves C. Li C. Li Y. Ge N. Montgomery E. (2018). Memristor-based analog computation and neural network classification with a dot product engine. *Adv. Mater.* 30:1705914. 10.1002/adma.201705914 29318659

[B7] Jang B. C. Nam Y. Koo B. J. Choi J. Im S. G. Park S. H. K. (2018). Memristive logic-in-memory integrated circuits for energy-efficient flexible electronics. *Adv. Funct. Mater.* 28:1704725. 10.1002/adfm.201704725

[B8] Jiang J. Guo J. Wan X. Yang Y. Xie H. P. Niu D. M. (2017). 2D MoS_2_ neuromorphic devices for brain-like computational systems. *Small* 13:1700933. 10.1002/smll.201700933 28561996

[B9] Kim B. Y. Hwang H. G. Woo J. U. Lee W. H. Lee T. H. Kang C. Y. (2017). Nanogenerator-induced synaptic plasticity and metaplasticity of bio-realistic artificial synapses. *NPG Asia Mater.* 9 e381–e381. 10.1038/am.2017.64

[B10] Kim B. Y. Lee W. H. Hwang H. G. Kim D. H. Lee S. H. Nahm S. (2016). Resistive switching memory integrated with nanogenerator for self-powered bioimplantable devices. *Adv. Funct. Mater.* 26 5211–5221. 10.1002/adfm.201505569

[B11] Kim S. Du C. Sheridan P. Ma W. Choi S. H. Lu W. D. (2015). Experimental demonstration of a second-order memristor and its ability to biorealistically implement synaptic plasticity. *Nano Lett.* 15 2203–2211. 10.1021/acs.nanolett.5b00697 25710872

[B12] Kumar M. Singh R. Kang H. Park J. Y. Kim S. Seo H. (2020). Brain-like spatiotemporal information processing with nanosized second-order synaptic emulators “solid-state memory visualizer”. *Nano Energy.* 76:105014. 10.1016/j.nanoen.2020.105014

[B13] Lee B. Cho H. Park K. T. Kim J. S. Park M. Kim H. (2020). High-performance compliant thermoelectric generators with magnetically self-assembled soft heat conductors for self-powered wearable electronics. *Nat. Commun.* 11 1–12. 10.1038/s41467-020-19756-z 33230141 PMC7684283

[B14] Lee T.-H. Hwang H.-G. Jang S. Wang G. Han S. Kim D.-H. (2017). Low-Temperature-Grown KNbO_3_ thin films and their application to piezoelectric nanogenerators and self-powered reram device. *ACS Appl. Mater. Interfaces* 9 43220–43229. 10.1021/acsami.7b11519 29144121

[B15] Li C. Belkin D. Li Y. Yan P. Hu M. Ge N. (2018). Efficient and self-adaptive in-situ learning in multilayer memristor neural networks. *Nat. Commun.* 9 1–8. 10.1038/s41467-018-04484-2 29921923 PMC6008303

[B16] Li Y. Li X. Fu L. Chen R. Wang H. Gao X. (2018). Effect of interface layer engineering on resistive switching characteristics of ZrO_2_-based resistive switching devices. *IEEE Trans. Electron Devices.* 65 5390–5394. 10.1109/TED.2018.2876942

[B17] Liu K. Yang P. Li S. Li J. Ding T. Xue G. (2016). Induced potential in porous carbon films through water vapor absorption. *Angew. Chem.-Int. Edit.* 55 8003–8007. 10.1002/anie.201602708 27159427

[B18] Liu S. Wang Y. Fardad M. Varshney P. K. (2018). A memristor-based optimization framework for artificial intelligence applications. *IEEE Circuits Syst. Mag.* 18 29–44. 10.1109/MCAS.2017.2785421

[B19] Liu Y. Li E. Wang X. Chen Q. Zhou Y. Hu Y. (2020a). Self-powered artificial auditory pathway for intelligent neuromorphic computing and sound detection. *Nano Energy* 78:105403. 10.1016/j.nanoen.2020.105403

[B20] Liu Y. Yang W. Yan Y. Wu X. Wang X. Zhou Y. (2020b). Self-powered high-sensitivity sensory memory actuated by triboelectric sensory receptor for real-time neuromorphic computing. *Nano Energy* 75:104930. 10.1016/j.nanoen.2020.104930

[B21] Liu Y. Zhong J. Li E. Yang H. Wang X. Lai D. (2019). Self-powered artificial synapses actuated by triboelectric nanogenerator. *Nano Energy* 60 377–384. 10.1016/j.nanoen.2019.03.079

[B22] Lübben M. Cüppers F. Mohr J. Witzleben M. Breuer U. Waser R. (2020). Design of defect-chemical properties and device performance in memristive systems. *Sci. Adv.* 6:9079. 10.1126/sciadv.aaz9079 32548248 PMC7272230

[B23] Luo W. Khoo Y. S. Kumar A. Low J. S. C. Li Y. Tan Y. S. (2018). A comparative life-cycle assessment of photovoltaic electricity generation in Singapore by multicrystalline silicon technologies. *Sol. Energy Mater. Sol. Cells.* 174 157–162. 10.1016/j.solmat.2017.08.040

[B24] MacVittie K. Katz E. (2014). Self-powered electrochemical memristor based on a biofuel cell-towards memristors integrated with biocomputing systems. *Chem. Commun.* 50 4816–4819. 10.1039/C4CC01540A 24687004

[B25] Ntinas V. Vourkas I. Abusleme A. Sirakoulis G. C. Rubio A. (2018). Experimental study of artificial neural networks using a digital memristor simulator. *IEEE Trans. Neural Netw. Learn. Syst.* 29 5098–5110. 10.1109/TNNLS.2018.2791458 29994426

[B26] Peng Y. Li S. W. Hu Z. Z. (2019). A self-learning dynamic path planning method for evacuation in large public buildings based on neural networks. *Neurocomputing* 365 71–85. 10.1016/j.neucom.2019.06.099

[B27] Prezioso M. Merrikh-Bayat F. Hoskins B. D. Adam G. C. Likharev K. K. Strukov D. B. (2015). Training and operation of an integrated neuromorphic network based on metal-oxide memristors. *Nature* 521 61–64. 10.1038/nature14441 25951284

[B28] Qi M. Cao S. Yang L. You Q. Shi L. Wu Z. (2020). Uniform multilevel switching of graphene oxide-based RRAM achieved by embedding with gold nanoparticles for image pattern recognition. *Appl. Phys. Lett.* 116:163503. 10.1063/5.0003696

[B29] Sakib M. N. Hassan R. Biswas S. N. Das S. R. (2017). Memristor-based high-speed memory cell with stable successive read operation. *IEEE Trans. Comput. Aided Des. Integr. Circuits Syst.* 37 1037–1049. 10.1109/TCAD.2017.2729464

[B30] Shao H. Wen Z. Cheng P. Sun N. Shen Q. Zhou C. (2017). Multifunctional power unit by hybridizing contact-separate triboelectric nanogenerator, electromagnetic generator and solar cell for harvesting blue energy. *Nano Energy.* 39 608–615. 10.1016/j.nanoen.2017.07.045

[B31] Stewart J. W. Vella J. H. Li W. Fan S. Mikkelsen M. H. (2020). Ultrafast pyroelectric photodetection with on-chip spectral filters. *Nat. Mater.* 19 158–162. 10.1038/s41563-019-0538-6 31768011

[B32] Sun Y. Zheng X. Yan X. Liao Q. Liu S. Zhang G. (2017). Bioinspired tribotronic resistive switching memory for self-powered memorizing mechanical stimuli. *ACS Appl. Mater. Interfaces.* 9 43822–43829. 10.1021/acsami.7b15269 29160691

[B33] Tan H. Liu G. Yang H. Yi X. Pan L. Shang J. (2017). Light-gated memristor with integrated logic and memory functions. *ACS Nano.* 11 11298–11305. 10.1021/acsnano.7b05762 29028312

[B34] Tan H. W. Liu G. Zhu X. J. Yang H. L. Chen B. Chen X. X. (2015). An optoelectronic resistive switching memory with integrated demodulating and arithmetic functions. *Adv. Funct. Mater.* 27 2797–2803. 10.1002/adma.201500039 25786781

[B35] Tao Y. Li X. Wang Z. Xu H. Ding W. Ma J. (2017). Improved resistive switching reliability by using dual-layer nanoporous carbon structure. *Appl. Phys. Lett.* 111:183504. 10.1063/1.5003331

[B36] Tao Y. Li X. Xu H. Wang Z. Ding W. Liu W. (2018). Improved uniformity and endurance through suppression of filament overgrowth in electrochemical metallization memory with AgInSbTe buffer layer. *IEEE J. Electron Devices Soc.* 6 714–720. 10.1109/JEDS.2018.2843162

[B37] Tao Y. Wang Z. Xu H. Ding W. Zhao X. Lin Y. (2020). Moisture-powered memristor with interfacial oxygen migration for power-free reading of multiple memory states. *Nano Energy* 71:104628. 10.1016/j.nanoen.2020.104628

[B38] Tian Q. Zhang X. Zhao X. Wang Z. Lin Y. Xu H. (2020). Dual buffer layers for developing electrochemical metallization memory with low current and high endurance. *IEEE Electron Device Lett.* 42:1. 10.1109/LED.2020.3047837

[B39] Tseng Y. T. Chen I. C. Chang T. C. Huang J. C. Shih C. C. Zheng H. X. (2018). Enhanced electrical behavior from the galvanic effect in Ag-Cu alloy electrode conductive bridging resistive switching memory. *Appl. Phys. Lett.* 113:053501. 10.1063/1.5023527

[B40] Van G. M. Bohte S. (2017). Artificial neural networks as models of neural information processing. *Front. Comput. Neurosci.* 11:114. 10.3389/fncom.2017.00114 29311884 PMC5742181

[B41] Wang Y. Wu H. Xu L. Zhang H. Yang Y. Wang Z. L. (2020). Hierarchically patterned self-powered sensors for multifunctional tactile sensing. *Sci. Adv.* 6:eabb9083. 10.1126/sciadv.abb9083 32875115 PMC7438107

[B42] Wang Z. Joshi S. Savel’ev S. Jiang H. Midya R. Lin P. (2016). Memristors with diffusive dynamics as synaptic emulators for neuromorphic computing. *Nat. Mater.* 16 101–108. 10.1038/NMAT4756 27669052

[B43] Wang Z. Joshi S. Savel’ev S. Song W. Midya R. Li Y. (2018). Fully memristive neural networks for pattern classification with unsupervised learning. *Nat. Electron.* 1 137–145. 10.1038/s41928-018-0023-2

[B44] Wang Z. Li C. Lin P. Rao M. Nie Y. Song W. (2019b). In situ training of feed-forward and recurrent convolutional memristor networks. *Nat. Mach. Intell.* 1 434–442. 10.1038/s42256-019-0089-1

[B45] Wang Z. Li C. Song W. Rao M. Belkin D. Li Y. (2019b). Reinforcement learning with analogue memristor arrays. *Nat. Electron.* 2 115–124. 10.1038/s41928-019-0221-6

[B46] Wang Z. Zeng T. Ren Y. Lin Y. Xu H. Zhao X. (2020). Toward a generalized Bienenstock-Cooper-Munro rule for spatiotemporal learning via triplet-STDP in memristive devices. *Nat. Commun.* 11 1–10. 10.1038/s41467-020-15158-3 32198368 PMC7083931

[B47] Wang Z. Q. Xu H. Y. Li X. H. Yu H. Liu Y. C. Zhu X. J. (2012). Synaptic learning and memory functions achieved using oxygen ion migration/diffusion in an amorphous InGaZnO memristor. *Adv. Funct. Mater.* 22 2759–2765. 10.1002/adfm.201103148

[B48] Wu C. Kim T. W. Park J. H. Koo B. Sung S. Shao J. (2019). Self-Powered tactile sensor with learning and memory. *ACS Nano* 14 1390–1398. 10.1021/acsnano.9b07165 31747246

[B49] Xu S. Guo L. Sun Q. Wang Z. L. (2019). Piezotronic effect enhanced plasmonic photocatalysis by AuNPs/BaTiO_3_ heterostructures. *Adv. Funct. Mater.* 29:1808737. 10.1002/adfm.201808737

[B50] Xu Z. Wu C. Li F. Chen W. Guo T. Kim T. W. (2018). Triboelectric electronic-skin based on graphene quantum dots for application in self-powered, smart, artificial fingers. *Nano Energy.* 49 274–282. 10.1016/j.nanoen.2018.04.059

[B51] Xue G. Xu Y. Ding T. Li J. Yin J. Fei W. (2017). Water-evaporation-induced electricity with nanostructured carbon materials. *Nat. Nanotechnol.* 12 317–321. 10.1038/nnano.2016.300 28135262

[B52] Yan X. Pei Y. Chen H. Zhao J. Zhou Z. Wang H. (2019a). Self-assembled networked PbS distribution quantum dots for resistive switching and artificial synapse performance boost of memristors. *Adv. Mater.* 31:1805284. 10.1002/adma.201805284 30589113

[B53] Yan X. Zhao J. Liu S. Zhou Z. Liu Q. Chen J. (2018). Memristor with Ag-cluster-doped TiO_2_ films as artificial synapse for Neuroinspired computing. *Adv. Funct. Mater.* 28:1705320. 10.1002/adfm.201705320

[B54] Yan X. Zhao Q. Chen A. Zhao J. Zhou Z. Wang J. (2019b). Vacancy-induced synaptic behavior in 2D WS_2_ nanosheet–based memristor for low-power neuromorphic computing. *Small* 15:1901423. 10.1002/smll.201901423 31045332

[B55] Yang X. Xiong Z. Chen Y. Ren Y. Zhou Y. Pan F. (2020). A self-powered artificial retina perception system for image preprocessing based on photovoltaic devices and memristive arrays. *Nano Energy.* 78:105246. 10.1016/j.nanoen.2020.105246

[B56] Yang Z. Wu J. Li P. Chen Y. Yan Y. Zhu B. (2020). Resistive random access memory based on gallium oxide thin films for self-powered pressure sensor systems. *Ceram. Int.* 46 21141–21148. 10.1016/j.ceramint.2020.05.191

[B57] Yin J. Li X. Yu J. Zhang Z. Zhou J. Guo W. (2014). Generating electricity by moving a droplet of ionic liquid along graphene. *Nat. Nanotechnol.* 9 378–383. 10.1038/nnano.2014.56 24705513

[B58] Yoon J. H. Wang Z. Kim K. M. Wu H. Ravichandran V. Xia Q. (2018). An artificial nociceptor based on a diffusive memristor. *Nat. Commun.* 9 1–9. 10.1038/s41467-017-02572-3 29379008 PMC5788850

[B59] Zhang M. Yeow J. T. W. (2020). A flexible, scalable, and self-powered mid-infrared detector based on transparent PEDOT: PSS/graphene composite. *Carbon* 156 339–345. 10.1016/j.carbon.2019.09.062

[B60] Zhang X. Liu S. Zhao X. Wu F. Wu Q. Wang W. (2017). Emulating short-term and long-term plasticity of bio-synapse based on Cu/a-Si/Pt memristor. *IEEE Electron Device Lett.* 38 1208–1211. 10.1109/LED.2017.2722463

[B61] Zhao F. Cheng H. Zhang Z. Jiang L. Qu L. (2015). Direct power generation from a graphene oxide film under moisture. *Adv. Mater.* 27 4351–4357. 10.1002/adma.201501867 26088604

[B62] Zhao F. Wang L. Zhao Y. Qu L. Dai L. (2017). Graphene oxide nanoribbon assembly toward moisture-powered information storage. *Adv. Mater.* 29:1604972. 10.1002/adma.201604972 27862418

[B63] Zhao Q. Xie Z. Peng Y. Wang K. Wang H. Li X. (2020). Current status and prospects of memristors based on novel 2D materials. *Mater. Horiz.* 7 1495–1518. 10.1039/C9MH02033K

[B64] Zhao X. Wang Z. Li W. Sun S. Xu H. Zhou P. (2017). Photoassisted electroforming method for reliable low-power organic-inorganic perovskite memristors. *Adv. Funct. Mater.* 30:1910151. 10.1002/adfm.201910151

[B65] Zhao X. Xu H. Wang Z. Li Y. Liu Y. (2019). Memristors with organic-inorganic halide perovskites. *InfoMat* 1 183–210. 10.1002/inf2.12012

[B66] Zhou F. Zhou Z. Chen J. Choy T. Wang J. Zhang N. (2019). Optoelectronic resistive random access memory for neuromorphic vision sensors. *Nat. Nanotechnol.* 14 776–782. 10.1038/s41565-019-0501-3 31308498

[B67] Zhou G. Ren Z. Wang L. Wu J. Sun B. Zhou A. (2019). Resistive switching memory integrated with amorphous carbon-based nanogenerators for self-powered device. *Nano Energy* 63:103793. 10.1016/j.nanoen.2019.05.079

[B68] Zidan M. A. Strachan J. P. Lu W. D. (2018). The future of electronics based on memristive systems. *Nat. Electron.* 1 22–29. 10.1038/s41928-017-0006-8

